# The complete chloroplast genome of *Musa acuminata* var. *chinensis*

**DOI:** 10.1080/23802359.2020.1788462

**Published:** 2020-07-07

**Authors:** Huimin Feng, You Chen, Xiaoxiong Xu, Hongli Luo, Yaoting Wu, Chaozu He

**Affiliations:** a Hainan Key Laboratory for Sustainable Utilization of Tropical Bioresources, Hainan University, Haikou, Hainan, China; b Hainan Tropical Ocean University, Sanya, Hainan, China

**Keywords:** *M. acuminata* var. *chinensis*, chloroplast genome, Illumina sequencing, phylogenetic analysis

## Abstract

*Musa acuminata* var. *chinensis* is one of the most important wild banana species native to China which has huge potential breeding value by its cold tolerance and disease resistance. In this study, we first reported the complete chloroplast genome of *M. acuminata* var. *chinensis* and explore its phylogenetic position using a maximum likelihood phylogenetic tree. The chloroplast genome of *M. acuminata* var. *chinensis* is 170,402 bp in length and the overall GC content of the whole genome is 36.8%. It consisting of a pair of inverted repeat (IR, 35,320 bp) regions, a large single-copy (LSC, 88,870 bp) and a small single-copy (SSC, 10,900 bp). The chloroplast genome contained 112 genes, including 79 protein-coding genes, 29 tRNA genes, and 4 rRNA ribosomal genes. The most genes occur as a single copy, while 23 gene species occur in double copies. Phylogenetic analysis of 7 selected chloroplast genomes revealed that *M. acuminata* var. *chinensis* was closely related to *M. acuminata* ssp. *malaccensis*. The complete chloroplast genome of *M. acuminata* var. *chinensis* will greatly enhance precious gene resources for banana breeding programs in the future.

*Musa acuminata* Colla var. *chinensis* Häkkinen & Wang Hong belonging to *Musa*, Musacaea, is one of the most important wild banana species native to China which has huge potential breeding value due to its its cold tolerance and disease resistance. It is distributed northward along the China–Myanmar border and is found in river watersheds along these slopes, at altitudes between 300 and 800 m that tolerate slight frost damage. It also can grow under shaded tree cover. It also has basal, hermaphroditic female flowers and therefore self-pollination occurs before the floral bracts open (Häkkinen and Wang 2007). The information of chloroplast genomes has been extensively applied in understanding plant genetic diversity and evolution that are of great importance in breeding programs. The complete chloroplast genome of *M. acuminata* var. *chinensis* will greatly enhance precious gene resources for banana breeding programs in the future.

An individual of *M. acuminata* var. *chinensis* was collected from Yunnan, China (22°39.170′N, 103°03.763′E). Total genomic DNA was extracted from fresh young leaves using modified CTAB method (Doyle and Doyle 1987) and deposited at Hainan Tropical Ocean University (Accession number: M05). Purified total genomic DNA was used for sequencing. Whole-chloroplast genome sequencing was outsourced to Shanghai Majorbio Bio-pharm Technology Co., Ltd, on an Illumina Hiseq X Ten platform. Approximately 5.5 G clean reads were obtained and further assembled into contigs using MITObim v1.8 (Hahn et al. 2013) and SOAPdenovo v2.04 (Luo et al. 2012), with the reference genome of *M.acuminata* subsp. *malaccensis* (Genbank: HF677508.1) (Martin et al. 2013). The assembled chloroplast genome was mapped and annotated using the online program Organellar Genome Draw tool v1.2 (Lohse et al. 2013). The annotated chloroplast genomic sequence was submitted to Genbank under the accession number of MT593357.

The complete chloroplast genome of *M. acuminata* var. *chinensis* is 170,402 bp in length consisting of a pair of inverted repeat (IR, 35,320 bp) regions, a large single copy (LSC, 88,870 bp) and a small single copy (SSC, 10,900 bp). The chloroplast genome contained 112 genes, including 79 protein-coding genes, 29 tRNA genes, and 4 rRNA ribosomal genes. Most of the genes occur as a single copy, while 23 gene species occur in double copies, including 11 protein-coding genes (ndhA, ndhB, ndhH, rps7, rps12, rps15, rps19, rpl2, rpl23, ycf1, ycf2), 8 tRAN genes (trnI-CAU, trnV-GAC, trnI-GAU, trnA-UGC, trnR-ACG, trnN-GUU, trnL-CAA, trnH-GUG) and 4 rRNA genes (rrn16, rrn23, rrn4.5, rrm5). The overall GC content of the whole genome is 36.8% and the corresponding values in LSC, SSC and IR regions are 35.18%, 31.03% and 39.75%, respectively.

A total of six additional complete chloroplast genomes of the family Musaceae were used to clarify the phylogenetic position of *M. acuminata* var. *chinensis. Musalla lasiocarpa* (KY807173) (Zhang et al. 2018) was used as the outgroup. All of the chloroplast genomes were aligned in MAFFT (Katoh and Standley 2013) and a maximum likelihood tree was constructed in IQTREE v1.6 (Nguyen et al. 2015; Hoang et al. 2018) based on TVM + F + I with 1000 bootstrap replicates (Figure 1). The phylogenetic tree reveals that *M. acuminata* var. *chinensis* was closely related to *M. acuminata* ssp. *malaccensis* compared to other species of *Musa*. The result provided valuable information for further banana breeding program.

**Figure 1.**
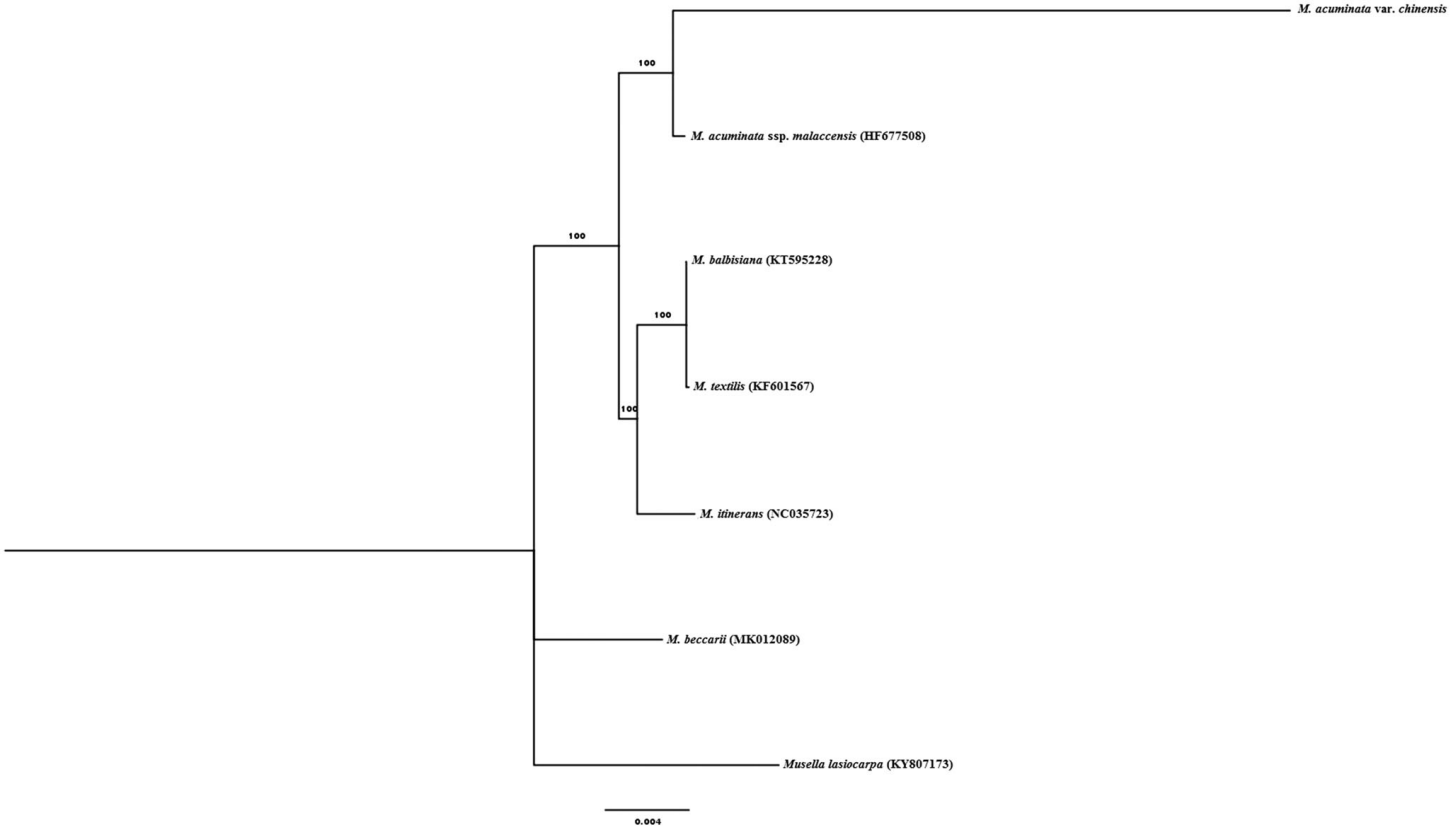
Maximum likelihood tree based on 7 complete chloroplast genomes of Musacaea.

## Data Availability

The data that support the findings of this study are openly available in national center for biotechnology information (NCBI) at https://www.ncbi.nlm.nih.gov/, reference number [MT593357].
